# Comparative Characteristics and Pathogenic Potential of *Escherichia coli* Isolates Originating from Poultry Farms, Retail Meat, and Human Urinary Tract Infection

**DOI:** 10.3390/life12060845

**Published:** 2022-06-06

**Authors:** Jolanta Sarowska, Tomasz Olszak, Agnieszka Jama-Kmiecik, Magdalena Frej-Madrzak, Bozena Futoma-Koloch, Andrzej Gawel, Zuzanna Drulis-Kawa, Irena Choroszy-Krol

**Affiliations:** 1 Department of Basic Sciences, Faculty of Health Sciences, Wroclaw Medical University, Chalubinskiego 4, 50-368 Wroclaw, Poland; jolanta.sarowska@umw.edu.pl (J.S.); magdalena.frej-madrzak@umw.edu.pl (M.F.-M.); irena.choroszy-krol@umw.edu.pl (I.C.-K.); 2 Department of Pathogen Biology and Immunology, University of Wroclaw, Przybyszewskiego 63/77, 51-148 Wroclaw, Poland; tomasz.olszak@uni.wroc.pl (T.O.); zuzanna.drulis-kawa@uwr.edu.pl (Z.D.-K.); 3 Department of Microbiology, University of Wroclaw, Przybyszewskiego 63/77, 51-148 Wroclaw, Poland; bozena.futoma-koloch@uwr.edu.pl; 4 Division of Avian, Exotic, Fur and Laboratory Animal Diseases, The Faculty of Veterinary Medicine, Wroclaw University of Environmental and Life Sciences, Norwida 31, 50-375 Wroclaw, Poland; andrzej.gawel@upwr.edu.pl

**Keywords:** *Escherichia coli*, virulence genes, antimicrobial resistance, ExPEC, UTI, retail food, poultry farms, MLST

## Abstract

The pathogenicity of many bacterial strains is determined by the acquisition of virulence genes and depends on many factors. The aim of this study was to analyse the phylogenetic background, virulence patterns, and drug susceptibility of 132 *E. coli* isolates tested in the context of the ExPEC (Extraintestinal Pathogenic *E. coli*) pathotype and the correlation of these features with bacterial isolation source: food (retail meat), poultry farms (AFEC—Avian Faecal *E. coli*), and patients with UTI (urinary tract infection) symptoms. The drug-susceptibility results of tested *E. coli* isolates obtained indicate that the resistance profile—ampicillin/tetracycline/trimethoprim+sulfamethoxazole/ciprofloxacin (AMP/TE/SXT/CIP)—was most frequently observed. The multidrug resistance (MDR) phenotype was found in 31.8% of isolates from poultry farms, 36.8% of strains isolated from food, and 20% of clinical samples. The greatest similarity of virulence profiles applied to isolates derived from poultry farms and food. Most of the AFEC from poultry farms and food-derived isolates belonged to commensals from phylogroups A and B1, while among the isolates from patients with UTI symptoms, the most common was the B2 phylogroup. The collective analysis showed similarity of the three studied groups of *E. coli* isolates in terms of the presented patterns of antimicrobial resistance, while the virulence profiles of the isolates studied showed great diversity. The phylogroup analysis showed no similarity between the poultry/food isolates and the UTI isolates, which had significant pathogenic potential.

## 1. Introduction

In the process of evolution, *E. coli* isolates acquire genes due to which they gain pathogenic potential, and the location of these genes within mobile genetic elements also enables their transfer to non-pathogenic *E. coli* isolates [1,2,3].

The presented research on the virulence and drug resistance comparison was carried out on *E. coli* isolates derived from patients with symptoms of UTI, from food and poultry farms isolated in the region of Lower Silesia in Poland (European Union). Since the problem of the rapid spread of virulent and drug-resistant bacteria affects both humans and animals, the action plan, in this case, is based on the ‘One Health’ approach [4]. The European Union (EU) member states during the World Health Assembly 2015 meeting committed to implementing the ‘One Health’ project, i.e., to combat and monitor antimicrobial resistance in humans, animals, and in food, and to promote high standards of cooperation in this field around the world. Increasing consumption and improper use of antibiotics, especially in non-hospital treatment, agriculture, and veterinary medicine, as well as in animal husbandry, causes rapid emergence and dissemination of bacterial drug resistance [5].

*Escherichia coli*, inhabiting mainly water environments, as well as belonging to animal and the human microbiome, can be equipped with a variety of virulence factors such as adhesins, toxins, iron acquisition systems, and other mechanisms enabling the avoidance of the host’s immune response [6]. ExPEC (Extraintestinal Pathogenic *E. coli*) are relative pathogens originating from the normal intestinal flora, responsible for parenteral infections among which the following groups of pathotypes are distinguished: uropathogenic (UPEC—Uropathogenic *E. coli*), associated with neonatal meningitis (NMEC—Neonatal meningitis *E. coli*), a sepsis-related group (SEPEC—Human sepsis-associated *E. coli*), and avian isolates (APEC—Avian Pathogenic *E. coli*). The ExPEC pathotype differs from commensals by the presence of functional genes which enable the colonization of a specific host organism. Within the pathogenicity islands present in the ExPEC reference strains (serotypes: O6: K2: H1, O6: K15: H31, O18: K1: H7, OR: K5: H, and O1: K1: H7), various iron uptake systems are coded, the most often mentioned being: *ent* (enterobactin), *iro* (salmochelin), *chu* (hemin uptake system), *sie* (iron and manganese transport), *iutA* (aerobactin), *fyuA* (yersiniabactin), adhesins: fimbriae *fim* (type 1), *pap* (P), *sfa* (S), *foc* (F1C), or toxins: *hly* (α-haemolysin), *CNF1* (cytotoxic necrosis factor), *vat* (vacuolating autotransporter toxin), or *sat* (autotransporter), but the spectrum of virulence genes detected for these isolates is much wider and varies with the isolate [7,8,9]. In turn, the most common APEC virulence genes determine the production of colicins (*cva*), aerobactin (*iut*), hemagglutinin (*tsh*), envelope antigens (*kps*), the *irp2* gene associated with iron transport genes, the *pap* operon encoding P-type fimbria, and the Iss protein associated with bactericidal resistance to the serum, encoded by the *iss* gene located within the plasmid pColV-I-K94 [10,11,12]. Johnson et al. describe five plasmid-borne genes as most significantly associated with APEC isolates: *iutA, hlyF, iss, iroN*, and *ompT* [13].

The analysis of the degree of polymorphism and phylogeny of isolates with ExPEC potential is an important subject for both researchers and clinicians, as it enables the monitoring of the emergence of new pathogenic isolates [14,15,16,17,18]. In recent years, a significant increase in antibiotic resistance has been observed, not only among the pathogenic but also commensal *E. coli* isolates. The prolonged exposure to antibiotics and simultaneously to different types of drugs used temporarily or on a huge scale in animal breeding leads to the selection of multi-resistance [19,20]. The control of ExPEC infections is a critical public health concern, particularly as ExPEC isolates harbour MDR genes. There is also the potential for ExPEC isolates to transfer these resistance genes to human-specific E. coli or other pathogenic bacteria. Monitoring to track ExPEC transmission and the associated AMR profiles in poultry farms would be important.

Our study aimed to report the prevalence of different virulence factors in the context of the ExPEC pathotype and drug resistance in *E. coli* isolates originated from poultry faeces and rectal swabs (poultry farms), retail meat (food), and human urinary tract infection (UTI).

These isolates were tested for the presence of eight ExPEC virulence genes (*fimH, papC, iha, usp, vat, pic, irp2, iss*) encoding adhesins, toxins, protectins, and iron uptake systems. Moreover, the drug sensitivity profile was also determined. In the second stage of the study, the relationships between the tested isolates were analysed based on genotyping methods such as the ERIC-PCR (Enterobacterial Repetitive Intergenic Consensus Chain Reaction Polymerase Chain Reaction) and multilocus sequence typing (MLST).

## 2. Materials and Methods

### 2.1. Escherichia coli Isolates

The isolates used in this study originated from three sources. The first group consisted of 44 isolates derived from chicken faeces and cloacal swabs collected from different types of poultry farms (cage, deep litter, free-range, and deep litter and free-range). The farms consisted of 1 to 11 poultry houses. The number of birds in the examined flocks ranged from 100 individuals in free-range systems to 57,000 individuals in cage systems (Table S1).

The farming system was classified as a cage system if birds were kept indoors in the cages, as deep litter if birds were kept indoors on litter, and as deep litter and free-range if birds were kept indoors on litter, but birds have access to the pasture during the sunny days and were closed in the building at night and during bad weather. The free-range was when birds were free to access the pastures every day.

From each bird flock kept in cage systems, 10 pooled faecal samples were collected, and from free-range and deep litter systems, cloacal swabs from 10 birds were taken. All samples were suspended 1/10 (*w/v* in buffered peptone water and cultured overnight at 37 °C. Each sample was cultured on MacConkey agar (Argenta, Poznan, Poland) and incubated at 37 °C for 24 h. Gram-negative, lactose-positive rods were identified using the ID 32E test on the ATB automated system. The obtained pure E. coli isolates cultures were stored in TSB liquid medium (Tryptic Soy Broth, BBL, Becton Dickinson, Sparks, Md.) with the addition of 15% glycerol at −80 °C.

The second pool included 38 isolates isolated from various types of raw meat (chicken, turkey, pork, beef) from retail sales in Wroclaw in 2015–2016 (Table S2). The Hazard Analysis and Critical Control Points System (HACCP) procedures are implemented in the food chain production, trade, and retail institutions, which regulate and protect food products against external microbiological contamination. To eliminate possible human origin contamination events, we only took vacuum-packed meat in a mechanical system.

The third group consisted of 50 clinical isolates derived from various cases of urinary tract infections in humans. *E. coli* isolates were derived from the urine samples of outpatients with UTI symptoms in the microbiology laboratory at the Department of Microbiology at the Medical University of Wroclaw between the period from 2015 to 2016. Patients were qualified for the microbiological examination based on clinical symptoms and urinary tract infection indicators. The study group included 50 people aged 15 to 90 years (average age—51 years), as shown in Table S3. Most of the E. coli isolates came from women, which constituted 66% of all the isolates tested in this group. E. coli isolates were most often derived from women in the age group over 66 years and in the age group 31–48 years, which constituted 22% and 18%, respectively. Among men, as in the case of women, the tested microorganisms were most often isolated from patients in the age group 31–48 years and over 66 years of age, and this percentage was 12% and 6%, respectively.

The positive control strain for all virulence tests was E. coli CFT 073 (ATCC 700928), a uropathogenic human pathogen producing adhesins: Type 1 Pili (fim), P (pap), S (sfa), F1C (foc), toxins: α-hemolysin (hly), vacuolating autotransporter toxin (vat), secreted autotransporter toxin (sat), and various iron acquisition systems [15]. Negative controls were two genetically modified strains: *E. coli* BL21(DE3) (Novagen, Pruszków, Poland; Sigma-Aldrich, Burlington, MA, USA) and *E. coli* TOP10 (Thermo-Scientific, Waltham, MA, USA). Both strains were artificially deprived of most virulence factors due to their conventional use in the cloning process. In addition, control tests were also performed using strain *E. coli* ATCC 8739, routinely used in medical and industrial quality control.

All bacterial isolates were stored at −80 °C in TSB medium (Trypticase Soy Broth, Biomerieux, Marcy-l’Étoile, France) with an addition of 10% DMSO (Chempur, Piekary Śląskie, Poland).

Primers for all PCR reactions were provided by Genomed (Warsaw, Poland). The reactions were carried out with the Eppendorf Mastercycler Nexus X2 and Nexus GX2e. Both the standard Taq polymerase (DreamTaq) and the other reagents were supplied by Thermo-Scientific. The reaction products were then electrophoretically separated in 1% agarose gel (Amresco, Solon, OH, USA) and visualized with Biorad Gel Doc XR. The band sizes were estimated in comparison to the GeneRuler 1 kb Plus and/or GeneRuler 100 bp mass markers (Thermo-Scientific).

### 2.2. Phylogenetic Groups of E. coli

To assess the genetic diversity, phylogenetic groups of the tested *E. coli* isolates were determined by a quadruplex-PCR method [16] with primers targeting the genes *chuA* (288 bp), *yjaA* (211 bp), *arpA* (400 bp), and a genetic region with an unknown function, TspE4C2 (152 bp). In the case of inconclusive results, for strains belonging to groups D/E or A/C, the PCR reaction was performed to detect the *arpA* (301 bp) and *trpA* (219 bp) genes. For the reaction, 25 µL of PCR mixture was set up per sample, containing 50 ng of template DNA, 2-U Taq polymerase DNA DreamTaq™ Green (Thermo Scientific, Vilnius, Lithuania), 2.5 µL of 10 × DNA DreamTaq™ Green Buffer (Thermo Scientific, Vilnius, Lithuania), 200-mM dNTP (Thermo Scientific, Vilnius, Lithuania), and 20 pmol each of the primers (Genomed, Warszawa, Poland). PCR amplifications were performed with parameters as follows: 95 °C for 4 min and 30 cycles of denaturation (30 s, 95 °C), annealing (20 s, 59 °C for quadruplex and phylogroup C and 20 s, 57 °C for phylogroup E), extension steps (1 min, 72 °C), and final extension (10 min, 68 °C). The list of the primers used for the determination of the phylogenetic groups of *E. coli* is presented in Table S4.

### 2.3. Enterobacterial Repetitive Intergenic Consensus Polymerase Chain Reaction (ERIC-PCR)

The ERIC-PCR technique was used for the genetic fingerprinting of 132 *E. coli* isolates. The replication of intergenerational palindrome sequences was performed using the classical PCR reaction, with primers: 5′ATGTAAGCTCTCCTGGGGGGATTCAC3′ and 5′AAGTAAGTGACTGGGGGGGGCG3′. The reaction was carried out using DreamTaq polymerase (Thermo Scientific) and Eppendorf Nexus X2 thermocycler. The process was performed in a volume of 25 μL, including 2.5 μL of 10× Taq buffer, 2.5 μL of each dNTP Mix, 0.6 μL of forward and reverse primers, 1 μg of template DNA, 0.62 U of DreamTag DNA polymerase, and nuclease-free water. The reaction products were isolated with 1% agarose gel electrophoresis (Amresco) and visualized with GelDoc XR (Biorad). The phylogenetic relationship between tested strains was determined with BioNumerics 7.6.3. software (Sint-Martens-Latem, Belgium) using Dice coefficient (1% of band matching tolerance and 1% of data optimization) followed by UPGMA (unweighted pair group method with arithmetic mean) clustering method. The results were presented in the form of dendrograms. 

### 2.4. Multilocus Sequence Typing (MLST)

Genetic characterisation of selected *E. coli* isolates was performed using the MLST technique (according to Achtman scheme) including 7 housekeeping genes (*adk*, *fumC*, *gyrB*, *icd*, *mdh*, *purA*, and *recA*), following the procedure described by Wirth et al. [17]. The appropriate primer set and PCR reaction conditions were taken from the EnteroBase [18]. The template for the PCR reaction was purified bacterial DNA, isolated using the GeneJET™ Genomic DNA Purification Kit (Thermo Scientific, Waltham, MA, USA). The correctness of the reaction products was initially assessed by agarose gel electrophoresis, then the samples were sequenced in a commercial laboratory (Genomed, Warszawa, Poland). The sequencing results were compared with the resources of the PubMLST database [19]. As a final step, the data were analysed in the PHYLOViZ 2.0 software (Open Source, GNU GPL v3 licence, http://www.phyloviz.net, accessed on 4 May 2022) [20] and the results were presented as a phylogenetic tree created according to the neighbour-joining algorithm based on Hamming distance.

### 2.5. Detection of Selected Virulence Genes (VGs)

Bacterial isolates listed in Tables S1–S3 were tested for the presence of selected virulence genes (*fimH*, *iha*, iss, *irp2, papC, pic, usp*, and *vat)*. A standard PCR reaction was performed, the starters used for each reaction and the characteristics of the virulence genes (VG) are presented in Table 1. The reactions were carried out using the Eppendorf Nexus X2 thermocycler and DreamTaq polymerase (Thermo Scientific). The reaction products were then electrophoretically separated in 1% agarose gel (Amresco) and visualized with Biorad Gel Doc XR. The band sizes were estimated in comparison to the Gene Ruler 1 kb Plus mass marker (Thermo Scientific).

### 2.6. Determination of E. coli Susceptibility to Antibiotics and Chemotherapeutics

The drug susceptibility testing including an extended ESBL-type resistance detection and was performed by the diffusion-disk method on Mueller–Hinton agar (MHA) following EUCAST recommendations [26]. The following antibiotics were used: amoxicillin (10 μg), tetracycline (10 μg), trimethoprim/sulfamethoxazole (1.25/23.75 μg), ciprofloxacin (5 μg), piperacillin (100 μg), nitrofurantoin (300 μg), chloramphenicol (30 μg), amoxicillin/clavulanic acid (21/10 μg), cefuroxime (30 μg), gentamicin (10 μg), piperacillin/tazobactam (100/10 μg), cefotaxime (30 μg), ceftazidime (30 μg), meropenem (10 μg), imipenem (10 μg), amikacin (30 μg). To control the results, *E. coli* ATCC 25922 and *E. coli* ATCC 35218 were used as reference strains. The results were interpreted as recommended by EUCAST and National Reference Centre for Drug Susceptibility guidelines [26].

### 2.7. Statistical Analysis

The correlations between the presence of virulence genes (VGs) and three groups of sources were described using the logistic regression mixed models. The correlated nature of isolates derived from one sample (food, UTI, or poultry farms) was taken into account in this approach. The chi-squared test or Fischer’s test (if the expected numbers of observations were small) were used to evaluate the frequency of VG between on-MDR and MDR groups within each source. The null hypothesis assumed that the proportions in pairs of isolates (UTI–food, UTI–poultry farms, poultry farms–food) are equal. The alternative hypothesis was one-sided, assuming that the proportion in one group is higher or lower. To measure the strength of the associations for the cross-tabulation of VGs from each source the Phi coefficient was calculated. Multidrug resistance (MDR) means resistance or reduced sensitivity to at least one antibiotic from at least three different groups of antimicrobial agents. The level of statistical significance was *p* = 0.05. The analyses were performed using the PQStat v.1.8.0.

## 3. Results

### 3.1. Phylogenetic Groups of E. coli

**Figure 1:**
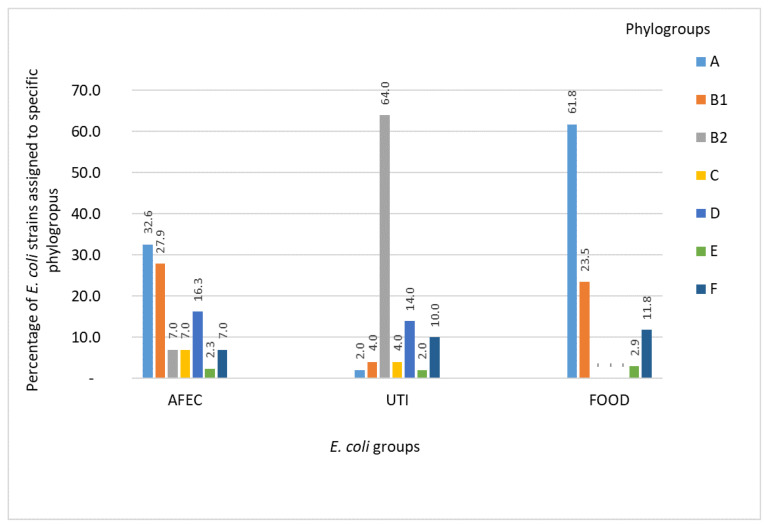
The percentage (%) share of *E. coli* (n = 132) isolates belonging to specific phylogroups. Note: AFEC—Poultry farms (n = 44), UTI—(n = 50) and FOOD—(n = 38).

The assignment of the AFEC, UTI, and FOOD strains to the respective phylogroups is presented in Figure 1 and Tables S5–S7. Among the AFEC isolates, 32.6% and 27.9% belonged to the nonpathogenic A and B1 groups, respectively, whereas 3 (7.0%) and 7 (16.3%) were assigned to the pathogenic B2 and D phylogroups, respectively. A high percentage of the UTI isolates belonged to the B2 phylogroup (64.0%; n = 32). The percentage shares of specific UTI isolates within the remaining phylogroups were as follows: 14.0% of the UTI isolates (n = 7) belonged to group D, 10.0% (n = 5) to group F, 4.0% (n = 2) to group B1, 4.0% (n = 2) to group C, 2.0% (n = 1) to group A, and 2.0% (n = 1) in group E. The percentage distribution of the phylogroups among the tested FOOD isolates was as follows: 61.8% (n = 21) of group A, 23.5% (n = 8) group B1, 11.8% (n = 4) group F, and 2.9% (n = 1) were in group E (Figure 1).

### 3.2. Characteristics of the Studied E. coli Isolates in the Context of Their Antibiotic Resistance Patterns and Virulence Profile

Based on the analysis of all isolates tested (n = 132), the highest level of resistance was observed for ampicillin (AMP) (43.2% Poultry farms, 47.4% FOOD, 38.0% UTI), tetracycline (TE) (29.5% Poultry farms, 47.4% FOOD, 34% UTI), trimethoprim/sulfamethoxazole (SXT) (22.7% Poultry farms, 34.2% FOOD, 30.0% UTI), and ciprofloxacin (CIP) (13.6% Poultry farms, 34.2% FOOD, 14% UTI) (Table S8). It was found that the most common resistance was the AMP/TE/SXT/CIP pattern present in eleven food isolates (Tables S9–S11).

**Figure 2:**
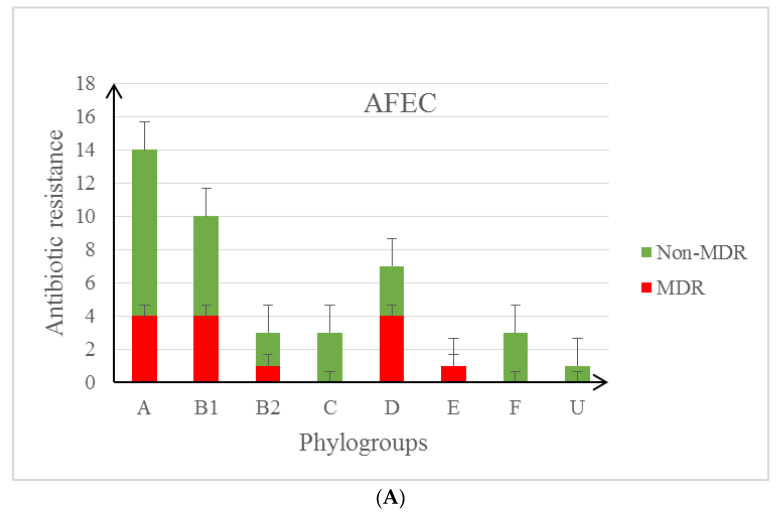

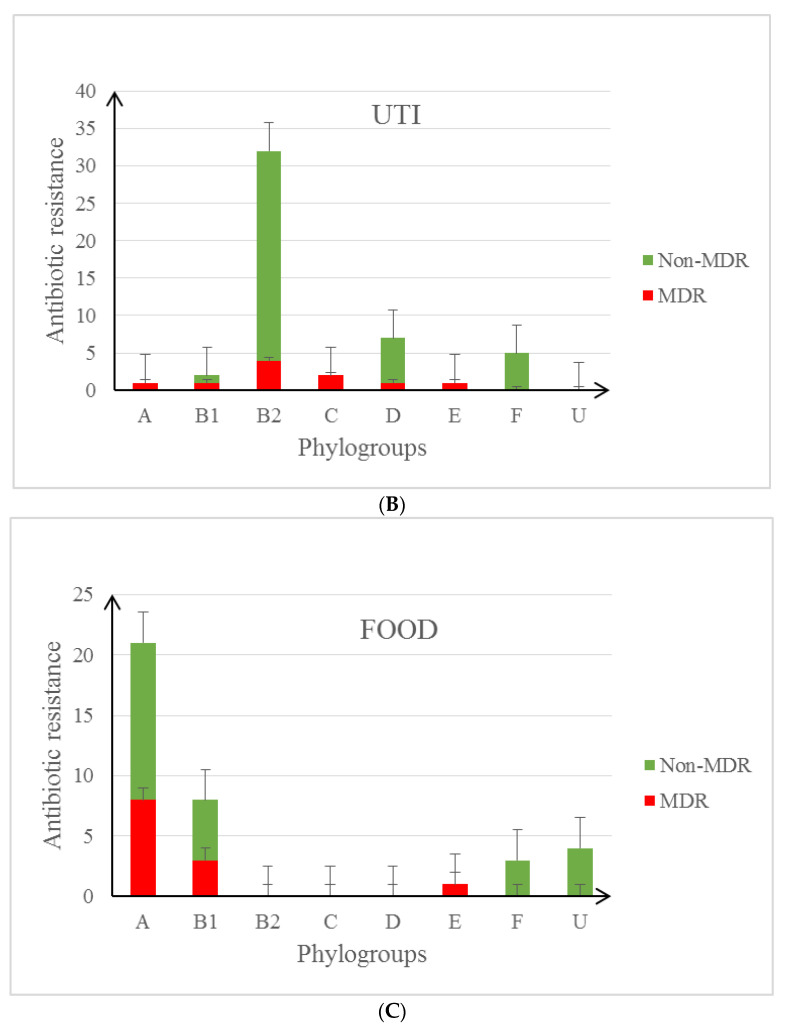
(**A**) Occurrence of MDR and Non-MDR resistance in individual phylogroups in AFEC strains. (**B**) Occurrence of MDR and Non-MDR resistance in individual phylogroups in UTI strains. (**C**) Occurrence of MDR and Non-MDR resistance in individual phylogroups in strains isolated from food.

The multidrug resistance (MDR—means resistance or reduced sensitivity to at least one antibiotic from at least three different groups of antimicrobial agents) phenotype was found in 31.8% of isolates from poultry farms (AFEC), 36.8% of isolates isolated from food, and 20.0% in UTI samples (Figure 2A–C and Tables S9–S11). Multidrug resistance was detected in 8/18 (MDR n = 4), 6/9 (MDR n = 6), 6/9 (MDR n = 4), 0/8 among *E. coli* isolates derived from poultry kept in a cage, deep litter, free-range and deep litter, and free-range method, respectively (Table S11). The ESBL phenotype was detected in only three cases (FD202 and FD3K2 isolated from a poultry farm and UTI 81) (Tables S9–S11).

**Figure 3:**
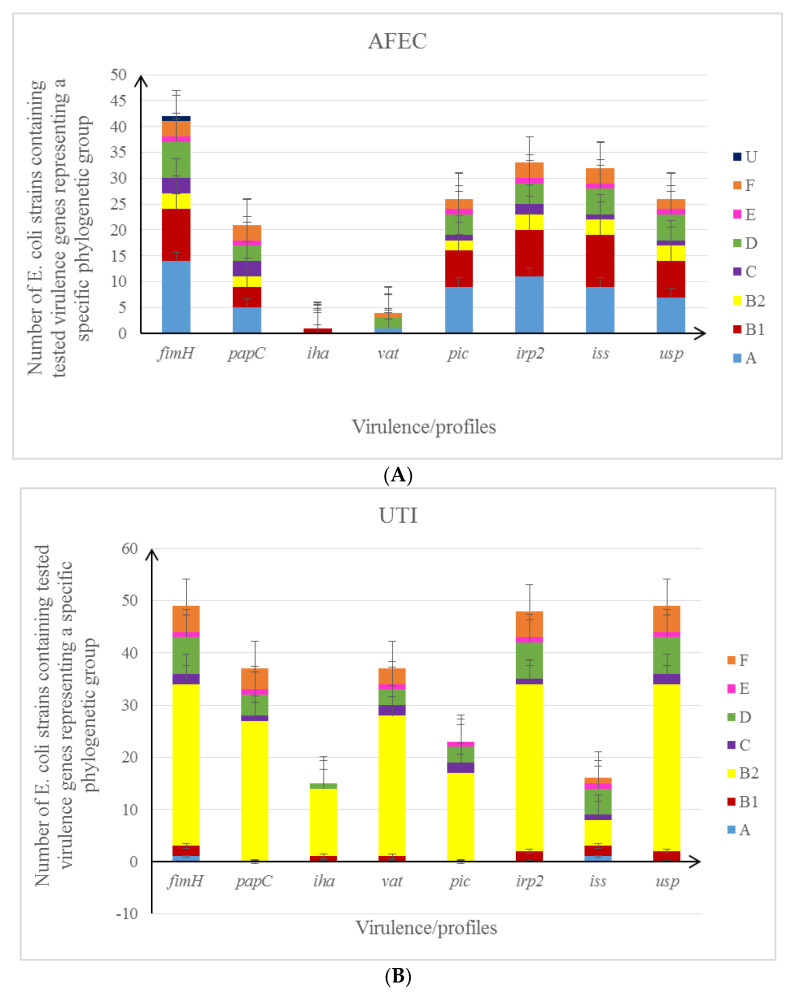

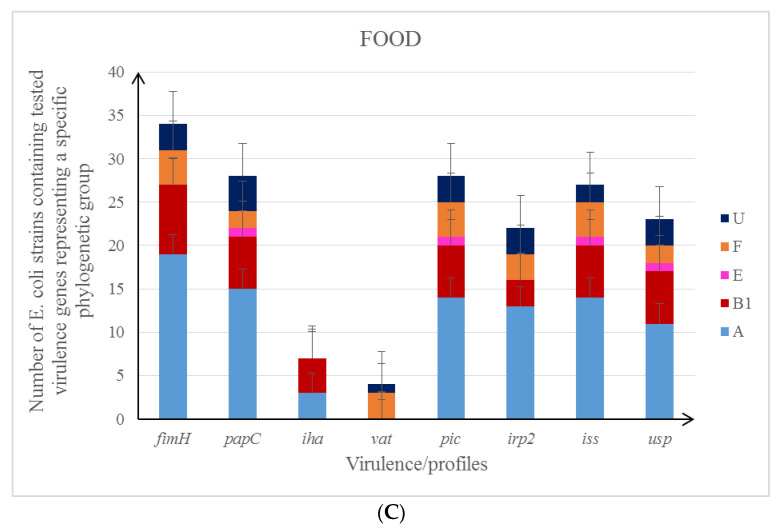
(**A**) (AFEC) The number of *E. coli* isolates containing tested virulence genes representing a specific phylogenetic group. (**B**) (UTI) The number of *E. coli* isolates containing tested virulence genes representing a specific phylogenetic group. (**C**) (FOOD) The number of *E. coli* isolates containing tested virulence genes representing a specific phylogenetic group.

The next step of the study was to characterise the pathogenicity potential of *E. coli* collection (*fimH/iha/iss/irp2/papC/pic/usp/vat*). Different combinations of genes detected in the tested isolates were referred to as virulence profiles (Figure 3A–C and Tables S9–S11). The greatest similarity of virulence profiles applied to isolates derived from poultry farms and food.

### 3.3. The Genotyping Analysis of E. coli Strains

The initial genotypic analysis and determination of phylogenetic relationships of tested *E. coli* isolates were carried out by the ERIC-PCR method. It allowed the grouping of 132 isolates into eleven ERIC clusters (ETI–ETXI) with similarity indices ranging from 92.4% to 100% (Figure S1). The greatest number of similarities in ERIC band patterns was seen in some isolates derived from poultry farms and food placed in six clusters: ETI-ETII, ETIV, and ETVI-ETVIII (Table S12).

**Figure 4:**
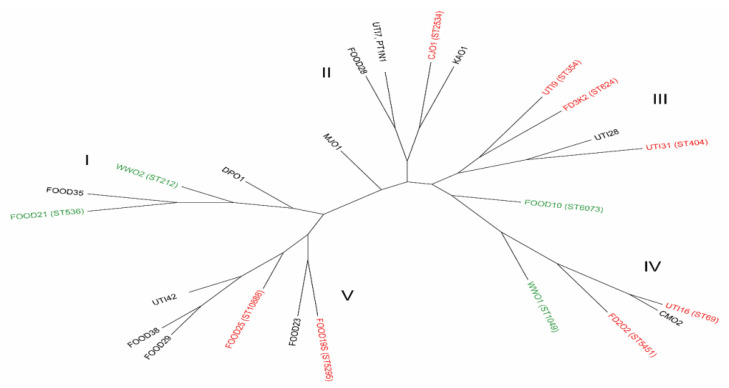
Phylogenetic tree (according to the neighbour-joining algorithm based on Hamming distance) was generated from the allelic profiles of seven housekeeping genes: adk, gyrB, fumC, icd, mdh, purA, and recA of 25 *E. coli* strains isolated from poultry farms, food, and patients with UTI symptoms. The sequence types (ST) highlighted in red (seven housekeeping genes) and sequence types highlighted in green (six housekeeping genes). I–V phylogenetic branches.

The second genotyping method used to determine the phylogenetic profiles was MLST. This method is recognized by clinicians and epidemiologists as the gold standard in bacterial genotyping [17] (Table S13). It was found that the tested *E. coli* isolates showed a high degree of genetic variation (12 sequence types among 25 isolates and 13 new ones) creating five (I-V) separate phylogenetic branches (Figure 4 and Table S14).

Comparing the two genotyping methods, we conclude that ERIC-PCR shows some divergence when compared to MLST. Based on ERIC-PCR analysis, the ST624, ST354, ST404, and ST5451 strains belong to the same cluster, while UTI28 (unknown ST) is in a separate cluster together with the ST536 strain (Figure 4 and Table S14). Based on the ERIC dendrogram (Figure S1, Tables S12 and S14), we could conclude that the ST69 and CM02 (unknown ST) strains show significant similarity (both belonging to the phylogroup D), while the remaining ST1049 andST6073 strains belong to different clusters.

In the case of strains collected within the phylogenetic branch marked with the symbol I (phylogroups A, B1, C), genetic relatedness was found only among strains from poultry farms (WW02 and ST212 and not described so far in the MLST DP01 database) and meat retail (FOOD 21 (ST536) and FOOD 35 (ST-unknown). Only one strain from poultry farms MJ01 belonging to the phylogroup C (unknown ST) was phylogenetically distinct and forms a separate tree line (Figure 4 and Table S14).

### 3.4. Statistical Analysis of the Obtained Results

In the next step of our study, we were statistically analysing the prevalence of different virulence patterns and drug resistance occurring in different groups of *E. coli* collection. In 132 *E. coli* isolates tested, derived from various sources, the incidence of individual virulence factors ranged from 2.3% to 98.0% (Table 2).

The conducted studies showed that the most frequently appearing virulence genes among strains isolated from patients with UTIs were *fimH* (98.0%), *usp* (98.0%), and *irp2* (96.0%), while the *iss* and *iha* genes had a lower incidence, respectively, at 32.0% and 28.0%. *Vat* and *papC* genes were detected in these bacteria at the same frequency of 74.0%, while the *pic* gene was detected much less frequently (46.0%)—Table 2.

In the food (F) and from poultry farms (P) strains, the most common amongst gene encoding adhesins was the *fimH* gene (89.5% F and 95.5% P), while *vat* (10.5% F and 9.1% P) and *iha* (13.1% F and 2.3% P) genes were detected with the lowest frequency, respectively. Based on the results obtained, it was also found that *E. coli* strains originating from poultry farms were characterized by a very high frequency (72.7%) of the *iss* gene detection, compared to isolates from the other two groups, as shown in Table 2.

Most of the genes (6 out of 8), occurred the most frequently in isolates from UTI, with statistically significant differences for: *fimH* (UTI compared to Food), *usp* (UTI compared to poultry farms), *vat* (UTI compared to food and to poultry farms). Two genes occurred significantly less frequently in the isolates of UTI. These were: *iss* (less frequent in UTI isolates compared to Poultry farms) and *pic* (less frequent in UTI compared to poultry farms and food)—Table 2.

Our results indicated that the *iss* and *pic* genes show the largest association (strong associations 0.5 < φ < 0.7) in a group of isolates derived from poultry farms (Table S15). On the other hand, in the group of isolates obtained from UTI, a strong association was observed for the *usp*, *irp2* genes (φ = 0.7), a moderate association for the *papC, irp2* (φ = 0.34) and the *vat, pic* genes (φ = 0.46), while the genes encoding the adhesins *papC* and *fimH* were weakly associated with the *iss* gene (respectively φ = 0.28, φ = 0.21). It confirms that the *iss* gene is more often related to APEC isolates (Table S16). Table S17 shows the dependence of virulence genes occurrence in food-derived *E. coli* isolates. A significant correlation was found for gene pairs *papC/pic* (*p* = 0.59), then *iss*/*pic* (*p* = 0.41), and the *iss*/*usp* (*p* = 0.40).

In multidrug-resistant (MDR) *E. coli*, the *iss* gene occurred significantly more frequently compared to drug-sensitive isolates (Poultry farms *p* = 0.04, UTI *p* = 0.04, Food *p* = 0.04) (Table 3).

## 4. Discussion

In recent years, a significant increase in antibiotic resistance has been observed not only among pathogenic but also commensal strains of E. coli. The increased use of antibiotics for therapeutic purposes and preventive measures in agriculture and animal husbandry has resulted in the spread of resistant bacteria [27]. The main threat to public health related to the production of food of animal origin, including poultry, is the possibility of human contamination with pathogenic microorganisms [28]. The source of drug-resistant strains may be animals involved in the transmission of microorganisms to humans through direct contact or indirectly through the food chain. Taking into account the groups of antibacterial agents distributed in Europe in 2016, which together accounted for 69.7% of veterinary drugs introduced for treatment, the largest group were tetracyclines (32.3%), penicillins (25.8%), and sulfonamides (11.6%) [29]. The effect of prolonged exposure to higher and higher concentrations of antibiotics is an increase in resistance, and the simultaneous exposure to different types of drugs leads to the selection of multiresistant strains. In gram-negative bacteria, antimicrobial resistance genes located on plasmids, transposons, or chromosomal DNA are often combined into integrons carrying genes encoding β-lactamases (bla) and genetic determinants that determine resistance to aminoglycosides, sulfonamides, tetracyclines, or quinolones. The integron gene cassettes detected in *E. coli* contain, among others, dfrA1, dfrA7 dihydrofolate reductase genes for trimethoprim resistance. and the bla, tet (B), and qnrB genes for resistance to β-lactam antibiotic, tetracycline, and quinolone [14]. Target enzyme modification causes resistance to metabolic pathway inhibitors such as trimethoprim/sulfamethoxazole [30]. *E. coli* strains with the ESBL phenotype are isolated from farm animals, broilers, pigs, and cattle [27]. In a French study conducted on 97 samples of chicken meat purchased in Lyon shopping centres, *E. coli* isolates showed high resistance to sulfonamides (84.4%), tetracycline (75.3%), trimethoprim (51.9%), quinolones (41.6%), aminoglycosides (29.9%), and phenicols (14.3%) [31]. Based on the results of our own research, it was found that the most common pattern of resistance in the studied *E. coli* isolates is AMP/TE/SXT/CIP, which was observed in 29% of food-derived isolates. The same pattern was found in 12% of isolates from UTI patients and 9% from poultry farms. A 2010 study in Germany analysed broiler meat samples isolates taken at slaughter, and most strains of *E. coli* isolated from the carcass and cecum were ESBL positive, at 88.6% and 72.5%, respectively [32]. Microorganisms that are dangerous to humans can also be transmitted through a contaminated environment. Studies published by Melendez et al. proved that ExPEC belonging to ST73 sequential was isolated from fresh faecal samples from wild marine mammals [33].

The initial molecular characterization of the isolates studied concerned the analysis of the presence and distribution of virulence factors in *E. coli* from various sources. ExPEC isolates owe their potential pathogenicity to the presence of various virulence factors that are responsible for colonization and defeat of host defence mechanisms. We searched selected virulence genes (*fimH/iha/iss*/*irp2/papC/pic/usp*/*vat*) for the ExPEC pathotype. It is currently known that only the interaction of many virulence factors necessary for the colonization of the host organism and related to the invasiveness of ExPEC, i.e., adhesins, toxins, or iron extraction systems, is associated with an increased probability of intestinal translocation [34,35]. The results of our study indicate that among 50 isolates from outpatients with UTI symptoms and 44 isolates obtained from poultry farms, these genes were detected with the following frequency, respectively: *iss*—32.0% and 72.7%, *papC*—74.0% and 52.3%, *fimH*—98.0% and 95.4%, and *irp2*—96.0% and 75.0%. The comparison of both groups of tested isolates confirmed the statistically significant similarity in terms of the *iss-papC-irp2* virulence profile with the ExPEC potential. In addition, several *E. coli* isolates from UTI patients and poultry farms were found to have the same virulence pattern: *fimH-usp-irp2-vat-papC-pic-iss*, which may indicate a similar pathogenic potential. As emphasized by other authors, the presence of the same *E. coli* isolates has been confirmed both in live animals and in food of animal origin. Food contamination with these isolates may lead to colonization of the human gastrointestinal tract, and under appropriate conditions may pose a potential risk of developing urinary tract infections [8,36,37]. Increased virulence potential is characterized by *E. coli* strains equipped with many genes coding for virulence factors, which primarily condition resistance to bactericidal action of serum (*iss*) and produce adhesins (*fimH, papC*) and bacterial toxins (*vat, pic*), which, as our research results show, were detected not only in strains isolated from patients with UTI symptoms, but also in others. Characterization of *E. coli* isolates obtained from poultry farms (different breeding methods) and isolated from meat samples (chicken, turkey, pork, beef) in the context of their antibiotic resistance and virulence profile allowed for very interesting observations. Particularly noteworthy is the fact that in the group of isolates from poultry farms, the highest levels of both virulence and antibiotic resistance were found in *E. coli* isolates obtained from cage-raised chickens. Italian studies have also shown that the use of alternative poultry farming methods that use less antibiotics result in a lower incidence of drug resistance among commensal *E. coli* isolates [38]. On the other hand, the analysis of virulence profiles and resistance patterns of food-derived isolates (different types of meat) indicates chicken meat with the highest pathogenic potential. Our study examined various types of meat, but as the MLST method showed, the greatest correlation was with *E. coli* isolates derived from poultry meat. It is worth noting that a higher proportion of MDR isolates derived from meat may suggest contamination at the stage of raw material preparation (food production).

Accurate determination of drug resistance and virulence profile for *E. coli* strains of ExPEC etiology may contribute to a better understanding of the mechanisms of pathogenesis of infections caused by these microorganisms [1,2,3]. The results obtained in our research indicate statistically significant differences in the frequency of virulence genes detected in the tested *E. coli* isolates studied in the context of their drug resistance profile, MDR and Non-MDR. It was found that the *iss* gene, which determines bactericidal resistance to complement, is more common in MDR strains, and this relationship applies to each of the studied groups of strains. MDR resistance was associated with a higher frequency of detection of these genes.

In 2011, research by Clermont et al. used the MLST method to study phylogenetic relationships and the presence of virulence genes for a group of *E. coli* strains isolated from various infections from humans and domestic animals, as well as from commensals belonging to this species. A thorough analysis of the study results showed that the etiological factor of parenteral infections was mainly strains belonging to the B2 group [34]. It was found that pathogenic *E. coli* strains isolated from animals and humans (the same type of infection) were closely related and shared many virulence genes. The observed differences concerned mainly the detected adhesin genes, which showed specificity for the host organism, but most interestingly, no such correlation was found in the B2 strains containing ExPEC pathogens [39]. The genes encoding adhesins are master genes that help bacteria colonize host tissues, which can lead to the development of infection. Type 1 fimbriae are the most common among UPEC strains. *E. coli* with type 1 fimbriae are more often isolated in cystitis because the natural receptor for these structures, UP 1a, is found on surface of the bladder mucosa. In turn, UPEC strains with P-type fimbriae are identified mainly in pyelonephritis. The FimH protein mediates not only bacterial adhesion, but also the invasion of bladder epithelial cells and is the most important virulence factor of UPEC strains. Recent studies also indicate the role of type 1 fimbriae in biofilm formation [40]. The frequency of these two genes in our *E. coli* collection was also high in strains isolated from patients with UTI symptoms (*fimH*-98.0% and *papC*-74.0%), food-derived isolates (*fimH*-89.5% and *papC*-73.7%) and from poultry farms (*fimH*-95.5% and *papC*-52.3%), which confirms the high adhesive potential of these strains. Another study found that most APEC isolates possessed one of the most important adhesin genes—*fimH* (96.0%), and with a lower frequency reaching 44.0%, the *papC* gene [39]. In kidney infection caused by UPEC, colonization is based on the system of mutual communication between type 1 and P fimbriae and consists of changing the expression of the *fim* and *pap* genes depending on the factors affecting the bacteria in the human body [41]. Taking into account the virulence profile of UPEC strains isolated from patients hospitalized in Mexico, most isolates were characterized by the ability to colonize the kidneys due to the high prevalence of the *papC* gene (62.0%) [42]. As shown by the results obtained by Ewers et al., as in UPEC, genes of the *pap* operon were detected with a high frequency (40.3%) also in APEC strains (sick chickens), but they were not found in any strain from the environment where these animals were kept [43]. Based on our own results, it was observed that although the *papC* gene was detected in *E. coli* strains from poultry farms, the method of breeding chickens influenced the presence of this trait in the studied isolates. In turn, the *iha* gene was detected with a low frequency in groups of strains from food farms and poultry, while in UTI isolates the presence of this gene was found at the level of 28.0%, which indicates greater adhesive capacity of strains responsible for infections. In the study by Johnson et al., the *iha* gene, which determines the synthesis of adhesin, was detected in patients with urosepsis at a frequency of 55.0% [44]. Based on our own results, it was observed that the incidence of adhesin coding genes was the highest for strains isolated from patients with UTI symptoms. Some UPEC virulence factors are closely related to a specific anatomical site of infection, e.g., the *iroN* and *usp* genes were most often detected in strains responsible for prostatitis [45]. The *usp* gene is located in the genomic DNA of uropathogenic *E. coli* strains as a component of small islands of pathogenicity and encodes Usp bacteriocin, which exhibits exonucleolytic activity, mainly consisting of the formation of pores in the cytoplasmic membrane and interfering with the synthesis of the cell wall [46].

In our study, the *usp* gene, as in the case of genes encoding adhesins, was most often detected in the group of isolates from UTI patients. Usp is an *E. coli* genotoxin that also acts on mammalian cells and can elicit apoptotic-specific responses. UPEC strains with this gene are often responsible for pyelonephritis, prostatitis, and urosepsis [47]. On the other hand, the literature data on the role of the iss gene in the pathogenesis of infections indicate that the presence of the iss gene is one of the virulence features of the APEC strains responsible for colibacillosis in poultry [48]. The results of the frequency of detection of the *iss* gene in isolates from poultry farms (72.7%) and food and retail meat (71.05%) obtained in our own research are consistent with those reported by McPeake et al. (72.8%) [49]. Additionally, in our study, a significantly lower incidence of the *iss* gene was observed in the case of isolates obtained from patients with UTI symptoms compared to *E. coli* strains isolated from both food and poultry farms, which may confirm that this is an example of a pathogenicity factor characteristic of APEC. The bacterial toxins analysed in our research were the Pic and Vat proteins, which belong to the SPATE family of autotransporters (serine protease autotransporters of the Enterobacteriaceae). They fall into two classes; cytotoxic transporters (e.g., Vat) belong to the former, and class II includes immunomodulators (e.g., Pic). Vat is the approximately 140 kDa virulence factor associated with urosepsis, which induces the formation of intracellular vacuoles, resulting in a cytotoxic effect similar to that caused by the VacA toxin found in *Helicobacter pylori*. The *vat* gene is encoded on the PAI pathogenicity island, while the *pic* gene is located on the bacterial chromosome [50,51]. The Pic protein has proteolytic activity, breaking down components of the complement system and mucins, but also having the ability to induce excessive mucus secretion. A statistically significant correlation coefficient for both of these genes, *vat* and *pic*, encoding ExPEC-related toxins, was found in the strains isolated from UTI patients. There was also a significant difference in the frequency of *vat* gene in *E. coli* strains isolated from patients with UTI symptoms (74.0%), and from food farms and poultry (10.5% and 9.1%, respectively), which indicates an increased potential for virulence of strains isolated from human infections. A similar relationship was observed in the study of Johnson et al., whose research material included 45 uropathogenic strains of *E. coli*, and the presence of the *vat* gene was detected in 31 isolates (68.0%) [52].

Determining the patterns of virulence and drug resistance of the studied isolates facilitates the observation of the relationship between them but does not confirm the hypothesis concerning their common source [2,11]. Many infections caused by MDR *E. coli* isolates are transmitted from person to person as a result of poor hygiene practices, but this does not preclude spreading through the food chain. Therefore, a very important issue in the case of suspected interspecies transmission is a thorough analysis of the phylogenetic background of the studied isolates [53].

Another objective of our research was to determine the degree of polymorphism and the relationship between the phylogeny of isolates with ExPEC potential. This ExPEC pathotype has also been previously reported in the world’s most common pandemic sequence types ST131, ST95, and ST73 from of the phylogenetic group B2 [33,54,55]. Genotyping methods are an excellent tool not only for the analysis of phylogenetic relationships and the spread of known pathogens but also for monitoring the emergence of new pathogenic isolates [16,18].

The results obtained by the MLST method showed a high degree of genetic variability of the studied *E. coli* isolates, which formed five separate phylogenetic branches not correlated with their origin. Our study showed the presence of an AFEC isolate in poultry farms, with sequence type ST624 described as ExPEC, and UTI (ST354) showing the ESBL phenotype. Both isolates belong to phylogroup B2 with confirmed presence of genes characteristic for the ExPEC pathotype (*usp*, *irp2*, and *iss*) and resistance to trimethoprim-sulfamethoxazole. The potential for global spread of MDR ST624, ST212, and ST1049 strains is also evidenced by the report from Colombia, where the ESBL/AmpC phenotype was found in both human and poultry samples [10]. In turn, studies conducted in Brazil confirmed the phylogenetic relationship of *E. coli* strains belonging to the sequence type ST354, isolated from hospitalized patients with UTI symptoms and chicken carcasses intended for retail sale. Epidemiological studies have confirmed the presence of *E. coli* isolates producing blaCMY-2 conjugated with ISEcp1, both in chickens and in humans in a specific area [56]. Sequential strains of *E. coli* type ST354 have also been found in various sources, such as birds, mammals, water samples, patients with urinary tract infections, and poultry meat samples [57]. Based on epidemiological studies carried out in Germany, it was proved that 7.1% of *E. coli* strains from UTI belonged to ST354 (inpatient and outpatient) with confirmed ExPEC markers in group F. These strains were responsible for parenteral infections in humans and animals, confirming the high potential of this type of sequences to spread in the widely understood environmental reservoir [58]. Avian and human ExPECs have similar virulence genes because they occupy similar niches in their hosts [1,59]. Specifically, both avian and human ExPECs must possess iron acquisition genes, among other virulence genes, due to the iron-poor environments they inhabit. Possessing similar virulence genes is not sufficient evidence of transmission. Even with this expected similarity in virulence genes, results of our data shows that the Urinary–Poultry comparisons were all statistically significant, meaning that they do not share the same virulence genes, again supporting the conclusion that the poultry isolates do not appear to be similar to the UTI isolates. Phylogenetic analysis carried out according to the Clermont scheme revealed considerable diversity among the *E. coli* isolates tested. Among the strains from patients with UTI symptoms, the B2 phylogroup was the most common, which along with D is considered a typical human ExPEC group. On the other hand, groups of isolates obtained from poultry farms (AFEC) and food were represented mainly by groups A and B1 related to commensal faecal strains. It is interesting that some AFECs were also assigned to parenteral pathogens (B2 and D), while the less frequently identified group F was found with a similar frequency in all studied groups of isolates. The phylogroup analysis showed no similarity between the poultry/food isolates and the UTI isolates.

It should be noted that accurate characterisation of the ExPEC feature should be constantly updated and tested in *E. coli* isolates isolated from different sources and geographic regions.

This requires the involvement of multiple research expert teams from vets, clinicians, microbiologists, food production industry, and animal breeders. The threat posed by the increase of antibiotic resistance determines the need to develop alternative antimicrobials, which could target specific determinants of the virulence of specific pathogens.

## 5. Conclusions

The pooled analyses of the data obtained in our study confirm the significant similarity of antimicrobial resistance patterns in the three studied groups of *E. coli* isolates obtained from food (retail meat), poultry farms, and those isolated from patients with UTI symptoms. In turn, the virulence profiles showed great diversity. In most UTI isolates, both virulence profiles and phylogeny confirm their ability to cause infections with the ExPEC pathotype. The phylogroup analysis showed no similarity between the poultry/food isolates and the UTI isolates. Our data have resulted in the conclusion that ExPEC pathotype transmission from poultry to humans is, at best, a rare occurrence.

## Figures and Tables

**Table 1:** Characteristics of virulence genes (VGs) used for PCR analysis.

Functions	Gene	Starter *Forward* (5′-3′) Starter *Reverse* (3′-5′)	Product Size (bp)
Adhesins	*fimH*—gene encoding for type 1 fimbria adhesin;	TGCAGAACGGATAAGCCGTGG GCAGTCACCTGCCCTCCGGTA	508 [21]
	*papC*—gene encoding for adhesin, an initiator of the formation of P-fimbria;	GTGGCAGTATGAGTAATGACCGTTA ATATCCTTTCTGCAGGGATGCAATA	205 [22]
	*iha*—gene encoding for adhesin homologous to the *Vibrio cholerae* receptor (IrgA);	CTGGCGGAGGCTCTGAGATCA TCCTTAAGCTCCCGCGGCTGA	827 [22]
Miscellaneous	*irp2*—gene encoding the protein responsible for iron acquisition, protectin;	AAGGATTCGCTGTTACCGGAC TCGTCGGGCAGCGTTTCTTCT	287 [23]
	*iss*—gene encoding protectin (increased serum survival gene);	CAGCAACCCGAACCACCTGATG AGCATTGCCAGAGCGGCAGAA	323 [24]
	*usp*—gene encoding a toxin which is a homologue of the *Vibrio cholerae* toxin;	CGGCTCTTACATCGGTGCGTTG GACATATCCAGCCAGCGAGTTC	615 [25]
Toxins	*vat*—gene encoding for the cytotoxin responsible for *E. coli* infection;	TCCTGGGACATAATGGTCAG GTGTCAGAACGGAATTGTC	981 [23]
	*pic*—gene encoding serine protease, toxin;	ACTGGATCTTAAGGCTCAGG TGGAATATCAGGGTGCCACT	409 [23]

**Table 2:** Frequency of virulence genes (functional categories) among *E. coli* isolates from UTI, food, and poultry farms.

Functional Cathegory VG	Number (%) of *E. coli* Isolates with VGs			
	UTI (N = 50)	FOOD (N = 38)	Poultry Farms (N = 44)	*p*-Value UTI/Food (U-F) UTI/Poultry Farms (U-P) Food/Poultry Farms (F-P)
Adhesins				
*fimH*	49 (98.0)	34 (89.5)	42 (95.4)	N.S.
*papC*	37 (74.0)	28 (73.7)	23 (52.3)	0.97 U-F 0.03 U-P * 0.04 F-P *
*iha*	14 (28.0)	5 (13.1)	1 (2.3)	0.14 U-F <0.01 U-P * 0.05 F-P *
Miscellaneous				
*irp2*	48 (96.0)	22 (57.9)	33 (75.0)	<0.01 U-F * 0.01 U-P * 0.05 F-P *
*iss*	16 (32.0)	27 (71.1)	32 (72.7)	<0.01 U-F * <0.01 U-P * 0.87 F-P
*usp*	49 (98.0)	23 (60.5)	26 (59.1)	<0.01 U-F * <0.01 U-P * 0.87 F-P
Toxins				
*vat*	37 (74.0)	4 (10.5)	4 (9.1)	<0.01 U-F * <0.01 U-P * 0.86 F-P
*pic*	23 (46.0)	28 (73.7)	25 (56.8)	0.01 U-F * 0.28 U-P * 0.12 F-P

* Statistically significant differences (ANOVA Test of statistical difference, Fisher posthoc test).

**Table 3:** Frequency of the VG (Non-MDR and MDR)—*E. coli* isolates from UTI, food, and poultry farms.

Number (%) of *E.coli* Isolates with VGs within Resistance Groups									
Functional Category VG	UTI (N = 50)			FOOD (N = 38)			Poultry Farms (N = 44)		
	Non-MDR (n = 40)	MDR (n = 10)	*p*	Non-MDR (n = 24)	MDR (n = 14)	*p*	Non-MDR (n = 30)	MDR (n = 14)	*p*
Adhesins									
*fimH*	40 (100.0) *	9 (90.0) *	0.04 *	21 (87.0)	13 (92.9)	0.98	29 (96.7)	13 (92.9)	0.83
*papC*	31 (77.5)	6 (60.0)	0.26	16 (66.7)	12 (85.7)	0.37	18 (60.0)	5 (35.7)	0.13
*iha*	12 (30.0)	3 (30.0)	1.0	3 (12.5)	4 (28.6)	0.42	1 (3.3)	0 (0.0)	0.69
Miscellaneous									
*irp2*	40 (100.0) *	8 (80.0) *	0.04	15 (62.5)	7 (50.0)	0.45	21 (70.0)	12 (85.7)	0.26
*iss*	10 (25.0) *	6 (60.0) *	0.03 *	13 (54.2) *	14 (100.0) *	0.01 *	19 (63.3) *	13 (92.8) *	0.04 *
*usp*	40 (100.0)	9 (90.0)	0.04 *	18 (75.0) *	5 (35.7) *	0.02 *	17 (56.7)	9 (64.3)	0.63
Toxins									
*vat*	29 (72.5)	8 (80.0)	0.63	3 (12.5)	1 (7.1)	0.69	2 (6.7)	2 (14.3)	0.80
*pic*	18 (45.0)	5 (50.0)	0.78	16 (66.7)	12 (85.7)	0.20	16 (53.3)	9 (64.3)	0.49

* Statistically significant differences in the frequency of VG between Non-MDR and MDR groups within one type are bolded—(Chi-Square test, Yate’s correction for continuity if expected frequencies <5).

## Data Availability

Data are contained within the article or supplementary material.

## Supplementary Materials

The following supporting information can be downloaded at: https://www.mdpi.com/article/10.3390/life12060845/s1, Tables S1–S17 and Figure S1: Dendrogram generated from ERIC-PCR banding pattern of 132 E. coli isolated from poultry farms, food and patients with UTI symptoms. The process was performed in a volume of 25 μL, including 2.5 μL of 10× Taq buffer, 2.5 μL of each dNTP Mix, 0.6 μL of forward and reverse primers, 1 μg of template DNA, 0.62 U of DreamTag DNA polymerase, and nuclease-free water. The reaction products were isolated with 1% agarose gel electrophoresis (Amresco) and visualized with GelDoc XR (Biorad). The similarity analysis was performed with Dice coefficient and UPGMA method.
