# Supplementary material for: Comparative Characteristics and Pathogenic Potential of Escherichia coli Isolates Originating from Poultry Farms, Retail Meat, and Human Urinary Tract Infection
Source: Life (Basel). 2022 Jun 6;12(6):845. doi: 10.3390/life12060845 (PMC9225339; doi:10.3390/life12060845)
Supplement: Supplementary file 1 [file life-12-00845-s001.zip › life-1735619-supplementary.pdf]

Supplementary Tables S1 – S17 and Figure S1

Table S1. *E. coli* strains isolated from poultry farms (Poultry farm)

#	Strain	Farming system	Flock size	Animals age (weeks)
1	BWO1	cage	30 000	36
2	BWO2	cage	30 000	36
3	BWO3	cage	30 000	36
4	CJO1	deep litter	800	80
5	CJO2	deep litter	800	80
6	CMK2	cage	1 500	22
7	CMO1	cage	1 500	22
8	CMO2	cage	1 500	22
9	DPO1	cage	5 000	30
10	DPO2	cage	5 000	30
11	DPO3	cage	5 000	30
12	FD1O1	deep litter	8 000	55
13	FD2O1	deep litter	10 000	29
14	FD2O2	deep litter	10 000	29
15	FD3K2	deep litter	6 000	10
16	FD3O1	deep litter	6 000	10
17	JAN1	free range	25 000	28
18	JAO1	free range	25 000	28
19	JAO2	free range	25 000	28
20	JAO3	free range	25 000	28
21	KAO1	free range	600	36
22	KAO2	free range	600	36

23	KBO1	free range	100	45
24	KBO2	free range	100	45
25	MJO1	cage	10 000	52
26	MJO2	cage	10 000	52
27	PT1K2	deep litter & free range	8 600	26
28	PT1N1	deep litter & free range	8 600	26
29	PT1N2	deep litter & free range	8 600	26
30	PT1O1	deep litter & free range	8 600	26
31	PT1O2	deep litter & free range	8 600	26
32	PT2K2	deep litter & free range	7 000	32
33	PT2O1	deep litter & free range	7 000	32
34	PT3K1	deep litter & free range	4 300	22
35	PT3K2	deep litter & free range	4 300	22
36	PWO1	deep litter	1 300	80
37	PWO2	deep litter	1 300	80
38	WWO1	cage	57 000	40
39	WWO2	cage	57 000	40
40	ZMK1M	cage	12 000	83
41	ZMN1M	cage	17 000	37
42	ZMN2M	cage	17 000	37
43	ZMN2S	cage	17 000	37
44	ZMN3M	cage	17 000	37

Table S2. *E. coli* strains isolated from food samples (FOOD)

#	Strain	Food sample
1	FOOD2	Chicken/breast
2	FOOD3	Chicken/breast
3	FOOD5	Chicken/breast
4	FOOD6	Chicken/breast

5	FOOD10	Chicken/breast
6	FOOD11	Chicken/breast
7	FOOD12	Pork/loin
8	FOOD13	Pork/loin
9	FOOD15	Pork/loin
10	FOOD16	Chicken/breast
11	FOOD18	Pork/loin
12	FOOD19	Pork/loin
13	FOOD20	Chicken
14	FOOD21	Chicken/breast
15	FOOD22	Chicken
16	FOOD23	Ground/turkey
17	FOOD24	Turkey/breast
18	FOOD25	Pork/shoulder
19	FOOD27	Ground/pork
20	FOOD28	Ground/chicken
21	FOOD29	Chicken/breast
22	FOOD30	Turkey
23	FOOD31	Chicken
24	FOOD33	Ground/chicken
25	FOOD34	Chicken
26	FOOD35	Turkey
27	FOOD36	Ground/pork
28	FOOD38	Chicken
29	FOOD41	Chicken
30	FOOD42	Chicken/wing
31	FOOD43	Pork/shoulder
32	FOOD44	Chicken
33	FOOD45	Chicken/leg

34	FOOD46	Chicken/wing
35	FOOD48	Chicken/breast
36	FOOD19S	Ground/beef
37	FOOD115N	Ground/pork
38	FOOD119N	Ground/beef

Table S3. *E. coli* strains isolated from patients with urinary tract infections (UTI)

#	Strain	Patient sex	Age
1	UTI1	female	44
2	UTI2	male	64
3	UTI3	male	41
4	UTI4	female	70
5	UTI5	female	87
6	UTI6	male	24
7	UTI7	female	90
8	UTI8	female	30
9	UTI9	female	41
10	UTI10	female	60
11	UTI11	female	63
12	UTI12	male	35
13	UTI13	female	34
14	UTI14	female	16
15	UTI15	female	15
16	UTI16	male	80
17	UTI17	male	34
18	UTI18	female	31
19	UTI19	female	70
20	UTI20	female	39

21	UTI21	male	40
22	UTI22	male	73
23	UTI23	female	86
24	UTI24	female	84
25	UTI25	female	66
26	UTI26	female	32
27	UTI27	male	60
28	UTI28	male	46
29	UTI29	female	32
30	UTI30	male	56
31	UTI31	female	38
32	UTI32	female	18
33	UTI33	male	16
34	UTI34	female	33
35	UTI35	female	33
36	UTI36	female	86
37	UTI37	female	62
38	UTI38	female	17
39	UTI39	female	28
40	UTI40	female	43
41	UTI41	female	73
42	UTI42	male	88
43	UTI43	female	84
44	UTI44	male	34
45	UTI45	female	82
46	UTI46	female	80
47	UTI47	male	65
48	UTI48	male	30
49	UTI49	female	23

50	UTI81	male	55

Table S4. The list of the primers (Genomed, Warszawa, Poland) used for the determination of the phylogenetic groups of *E. coli* in the quadruplex-PCR method

Gene	Sequence of Primers	PCR Product Size (bp)	Gene Bank ID
yjaA	5’ -CAAACGTGAAGTGTCAGGAG-3’<br>5’ -AATGCGTTCCTCAACCTGTG-3’	288	948515
chuA	5’ -CAAACGTGAAGTGTCAGGAG-3’<br>5’ -AATGCGTTCCTCAACCTGTG-3’	211	7155958
TspE4C2	5’ -CACTATTCGTAAGGTCATCC-3’<br>5’ -AGTTTATCGCTGCGGGTCGC-3’	152	EU240725.1
arpA	5’ -AACGCTATTCGCCAGCTTGC-3’<br>5’ -TCTCCCCATACCGTACGCTA-3’	400	7155679
arpA	5’ -GATTCCATCTTGTCAAAATATGCC-3’<br>5’-GAAAAGAAAAAGAATTCCCAAGAG 3’	301	944933
trpA	5’ -AGTTTTATGCCCAGTGCGAG-3’<br>5’ -TCTGCGCCGGTCACGCCC-3’	219	912862
trpA	5’ -CGGCGATAAAGACATCTTCAC-3’<br>5’ -GCAACGCGGCCTGGCGGAAG-3’	489	13702525

For the reaction, there was set up 25 µl of PCR mixture per sample containing 50 ng of template DNA, 2-U Taq polymerase DNA DreamTaq™ Green (Thermo Scientific, Vilnius, Lithuania), 2.5 µL of 10 × DNA DreamTaq™ Green Buffer (Thermo Scientific, Vilnius, Lithuania), 200-mM dNTP (Thermo Scientific, Vilnius, Lithuania) and 20 pmol each of the primers (Genomed, Warszawa, Poland). PCR amplifications were performed with parameters as follows: 95 °C for 4 min and 30 cycles of denaturation (30 s, 95 °C), annealing (20 s, 59 °C for quadruplex and phylogroup C and 20 s, 57 °C for phylogroup E), extension steps (1 min, 72 °C) and final extension (10 min, 68 °C).

Table S5. Characterization of *E. coli* isolates from poultry farms (Poultry farm – AFEC)

Strains	Ph	Genes amplified in quadruplex PCR reactions				group C	group E
		arpA	*chuA*	*yjaA*	*TspE4.C2*	*arpA (ArpAgpC)*	*arpA (ArpAgpE)*
BWO1	B1	+	-	-	+	+	-
BWO2	B2	-	+	+	-	-	-
BWO3	B1	+	-	-	+	+	-
CJO1	B1	+	-	-	+	-	-
CJO2	A	+	-	+	-	-	-
CMO1	B1	+	-	-	+	+	-
CMO2	D	+	+	-	+	+	-
CMK2	B1	+	-	-	+	+	-
DPO1	C	+	-	+	-	+	-
DPO2	B1	+	-	-	+	+	-
DPO3	A	+	-	+	-	-	-
FD1O1	A	+	-	-	-	+	-
FD2O1	A	+	-	-	-	-	-
FD2O2	D	+	+	-	-	+	-
FD3O1	A	+	-	-	-	-	-
FD3K2	B2	-	+	-	+	+	-
JAO1	F	-	+	-	-	+	-
JAO2	A	+	-	+	-	-	-
JAO3	A	+	-	-	-	-	-
JAN1	A	+	-	-	-	+	-
KAO1	A	+	-	-	-	-	-
KAO2	B1	+	-	-	+	+	-
KBO1	A	+	-	-	-	-	-
KBO2	A	+	-	-	-	-	-
MJO1	C	+	-	+	-	+	-

MJO2	A	+	-	-	-	-	-
PT1O1	B1	+	-	-	+	+	-
PT1O2	B1	+	-	-	+	+	-
PT1K2	B1	+	-	-	+	+	-
PT1N1	A	+	-	-	-	-	-
PT1N2	B1	+	-	-	+	-	-
PT2O1	D	+	+	-	-	+	-
PT2K2	D	+	+	-	-	+	-
PT3K1	D	+	+	-	-	+	-
PT3K2	E	+	+	-	-	+	+
PWO1	D	+	+	-	-	+	-
PWO2	C	+	-	+	-	+	-
WWO1	B1	+	-	-	+	+	-
WWO2	A	+	-	-	-	+	-
ZMK1M	U	+	-	+	+	+	-
ZMN1M	B2	-	+	+	-	+	-
ZMN2M	F	-	+	-	-	+	-
ZMN3M	D	+	+	-	-	+	-
ZMNS2	F	-	+	-	-	+	-

Ph – Phylo-group; U – Unknown;

Table S6. Characterization of *E. coli* isolates ambulatory patients with UTI symptoms (UTI)

Strains	Ph	Genes amplified in quadruplex PCR reactions				group C	group E
		arpA	*chuA*	*yjaA*	*TspE4.C2*	*arpA (ArpAgpC)*	*arpA (ArpAgpE)*
UTI 1	B2	-	+	+	-	-	-
UTI 2	B2	-	+	+	-	-	-
UTI 3	B2	-	+	+	-	-	-

UTI 4	B2	-	+	+	-	+	-
UTI 5	B2	-	+	+	+	+	-
UTI 6	B2	-	+	+	-	+	-
UTI 7	B1	+	-	-	+	+	-
UTI 8	B2	-	+	+	+	+	-
UTI 9	B2	-	+	-	+	+	-
UTI 10	D	+	+	-	-	+	-
UTI 11	B2	-	+	+	+	+	-
UTI 12	B2	-	+	+	+	+	-
UTI 13	B2	-	+	+	-	-	-
UTI 14	B1	+	-	-	+	-	-
UTI 15	F	-	+	-	-	-	-
UTI 16	D	+	+	-	-	+	-
UTI 17	B2	-	+	+	+	+	-
UTI 18	B2	-	+	+	+	+	-
UTI 19	B2	-	+	+	-	-	-
UTI 20	D	+	+	-	-	+	-
UTI 21	B2	-	+	+	-	-	-
UTI 22	B2	-	+	+	-	-	-
UTI 23	B2	-	+	+	-	+	-
UTI 24	B2	-	+	+	-	+	-
UTI 25	B2	-	+	+	+	+	-
UTI 26	F	-	+	-	-	+	-
UTI 27	F	-	+	-	-	+	-
UTI 28	F	-	+	-	-	-	-
UTI 29	B2	-	+	+	-	+	-
UTI 30	B2	-	+	+	-	-	-
UTI 31	B2	-	+	+	-	+	-
UTI 32	B2	-	+	+	-	+	-
UTI 33	B2	-	+	+	-	-	-

UTI 34	B2	-	+	+	+	+	-
UTI 35	E	+	+	-	-	+	+
UTI 36	D	+	+	-	-	+	-
UTI 37	B2	-	+	+	-	-	-
UTI 38	B2	-	+	+	+	+	-
UTI 39	F	-	+	-	-	-	-
UTI 40	B2	-	+	+	-	-	-
UTI 41	B2	-	+	+	-	-	-
UTI 42	C	+	-	+	-	+	-
UTI 43	B2	-	+	+	-	+	-
UTI 44	D	+	+	-	-	+	-
UTI 45	D	+	+	-	+	+	-
UTI 46	D	+	+	-	-	+	-
UTI 47	A	+	-	-	-	+	-
UTI 48	B2	-	+	+	+	-	-
UTI 49	B2	-	+	+	+	-	-
UTI 81	C	+	-	+	-	+	+

Ph – Phylo-group;

Table S7. Characterization of *E. coli* isolates from food (FOOD)

Strains	Ph	Genes amplified in quadruplex PCR reactions				group C	group E
		arpA	*chuA*	*yjaA*	*TspE4.C2*	*arpA (ArpAgpC)*	*arpA (ArpAgpE)*
FOOD 2	B1	+	-	-	+	+	+
FOOD 3	F	-	+	-	-	+	-
FOOD 5	A	+	-	-	-	-	-
FOOD 6	A	+	-	-	-	-	-
FOOD 10	B1	+	-	-	+	+	-

FOOD 11	F	-	+	-	-	+	-
FOOD 12	A	+	-	-	-	-	-
FOOD 13	A	+	-	-	-	-	-
FOOD 15	A	+	-	-	-	-	-
FOOD 16	U	-	-	+	+	-	-
FOOD 18	A	+	-	-	-	-	-
FOOD 19	A	+	-	-	-	-	-
FOOD 20	B1	+	-	-	+	+	-
FOOD 21	A	+	-	-	-	-	-
FOOD 22	A	+	-	-	-	-	-
FOOD 23	U	+	-	+	+	+	-
FOOD 24	A	+	-	-	-	-	-
FOOD 25	A	+	-	-	-	+	-
FOOD 27	B1	+	-	-	+	+	-
FOOD 28	B1	+	-	-	+	+	-
FOOD 29	U	+	-	+	+	+	-
FOOD 30	B1	+	-	-	+	-	-
FOOD 31	A	+	-	-	-	+	-
FOOD 33	A	+	-	-	-	+	-
FOOD 34	E	+	+	-	+	+	+
FOOD 35	B1	+	-	-	+	+	-
FOOD 36	F	-	+	-	-	-	-
FOOD 38	U	+	-	+	+	+	-
FOOD 41	B1	+	-	-	+	+	-
FOOD 42	F	-	+	-	-	+	-
FOOD 43	A	+	-	-	-	+	+
FOOD 44	A	+	-	-	-	-	-
FOOD 45	A	+	-	-	-	+	-
FOOD 46	A	+	-	+	-	-	-
FOOD 48	A	+	-	+	-	-	-

FOOD 19 S	A	+	-	-	-	+	-
FOOD 115N	A	+	-	-	-	+	-
FOOD 119N	A	+	-	+	-	-	-

Ph – Phylo-group; U – Unknown;

Table S8. Antibiotic resistance of *E. coli* strains isolated from various sources

Antimicrobials	Source (number of isolates)		
	Poultry farm (44) n (%)	FOOD (38) n (%)	Patients/UTIs (50) n (%)
AMP	19(43.2)	18(47.4)	19(38.0)
TE	13(29.5)	18(47.4)	17(34.0)
SXT	10(22.7)	13(34.2)	15(30.0)
CIP	6(13.6)	13(34.2)	7(14.0)
PRL	6(13.6)	1(2.6)	6(12.0)
C	6(13.6)	1(2.6)	2(4.0)
AMC	2(4.5)	0	2(4.0)
CXM	1(2.3)	0	1(2.0)
GE	1(2.3)	0	0
NF	0	0	2(4.0)
TZP	0	0	0
CTX	2(4.5)	0	0
CAZ	0	0	0
MEM	0	0	0
IPM	0	0	0
AK	0	1(2.6)	0

Note: AMP - Ampicillin 10, TE - Tetracycline 30, SXT - Trimethoprim-Sulfamethoxazole 1.25/23.75, CIP - Ciprofloxacin, PRL - Piperacillin 100, C - Chloramphenicol 30, AMC - Amoxicillin/clavulanic acid 20/10, CXM - Cefuroxime 30, GE - Gentamicin 10, NF - Nitrofurantoin 300, TZP - Piperacillin/Tazobactam 100/10, CTX - Cefotaxime 30, CAZ - Ceftazidime 30, MEM - Meropenem 10, IPM - Imipenem 10, AK - Amikacin 30;

Table S9. Genotypic and phenotypic characterization of E. coli isolates ambulatory patients with UTI symptoms (UTI)

No./ patient sex	Strains	Ph	Virulence/profiles								Resistance/patterns
			Adhesins			Toxins		Miscellanous			
			fimH	*papC*	*iha*	*vat*	*pic*	*irp2*	*iss*	*usp*	
1/F	UTI 40	B2	■			■	■	■		■	
2/F	UTI 10	D	■					■		■	AMP
3/F	UTI 11	B2	■		■			■		■	CIP
4/F	UTI 13	B2	■	■	■	■	■	■	■	■	
5/F	UTI 14	B1	■		■			■	■	■	TE
6/F	UTI 15	F	■			■		■	■	■	TE
7/F	UTI 18	B2	■	■	■	■	■	■		■	
8/F	UTI 19	B2	■	■			■	■		■	
9/F	UTI 20	D	■	■	■	■	■	■	■	■	
10/F	UTI 23	B2	■	■		■		■		■	
11/F	UTI 24	B2	■	■	■			■		■	AMP/TE/C
12/F	UTI 25	B2	■	■				■		■	
13/F	UTI 26	F	■	■				■		■	
14/F	UTI 29	B2	■	■		■	■	■		■	AMP/TE
15/F	UTI 31	B2	■	■		■	■	■		■	AMP/SXT
16/F	UTI 32	B2	■	■		■	■	■		■	AMP/SXT
17/F	UTI 34	B2	■	■	■	■	■	■		■	
18/F	UTI 35	E	■	■		■	■	■	■	■	AMP, TE, SXT, CIP, PRL
19/F	UTI 36	D	■	■		■	■	■	■	■	AMP/TE/SXT/CIP
20/F	UTI 37	B2	■	■		■		■		■	
21/F	UTI 38	B2	■	■		■		■		■	
22/F	UTI 39	F	■	■		■		■		■	
23/F	UTI 4	B2	■	■		■	■	■		■	
24/F	UTI 41	B2	■	■		■		■		■	TE
25/F	UTI 43	B2	■	■	■	■		■		■	AMP/TE/PRL
26/F	UTI 45	D	■	■		■	■	■	■	■	TE/SXT
27/F	UTI 46	D	■					■	■	■	AMP/PRL

28/F	UTI 49	B2	■		■	■		■	■	■	SXT
29/F	UTI 5	B2	■	■	■	■	■	■		■	
30/F	UTI 7	B1	■			■		■	■	■	AMP/TE/SXT/CIP
31/F	UTI 8	B2	■	■	■	■		■		■	
32/F	UTI 9	B2				■		■	■	■	AMP/TE/SXT
33/F	UTI 1	B2	■	■	■	■	■	■		■	SXT
n(%)	33(66.0)		32	24	12	25	15	33	11	33	MDR(n=6)
34/M	UTI 12	B2	■	■	■	■	■	■		■	AMP/AMC/SX/PRL
35/M	UTI 16	D	■					■		■	AMP/SXT
36/M	UTI 17	B2	■			■	■	■		■	
37/M	UTI 21	B2	■	■		■		■		■	
38/M	UTI 22	B2	■	■	■	■	■	■		■	NF
39/M	UTI 27	F	■	■				■		■	
40/M	UTI 28	F	■	■		■		■		■	AMP
41/M	UTI 3	B2	■	■		■	■	■		■	
42/M	UTI 30	B2	■	■		■		■	■	■	AMP
43/M	UTI 33	B2	■	■	■	■	■	■	■	■	
44/M	UTI 42	C	■	■		■	■	■	■	■	AMP/TE/SXT/CIP/NF
45/M	UTI 44	D	■	■				■	■	■	AMP/SXT
46/M	UTI 47	A	■						■		AMC/TE/SXT/CIP/PRL/C
47/M	UTI 48	B2	■	■		■		■		■	TE
48/M	UTI 6	B2	■	■				■		■	TE
49/M	UTI 81	C	■			■	■			■	TE/SXT/CIP/PRL/ESBL+
50/M	UTI 2	B2	■	■		■	■	■		■	AMP/TE
n(%)	17(34.0)		17	13	3	12	8	15	5	16	MDR(n=4)
Total n(%)	50 (100.0)		49 (98.0)	37 (74.0)	15 (30.0)	37 (74.0)	23 (46.0)	48 (96.0)	16 (32.0)	49 (98.0)	MDR total n=10 (20.0)

Patient

sex: F – female, M – male; Ph – Phylogroup; U – Unknown

Note: MDR–multidrug resistance; AMP-ampicillin, TE-tetracycline, SXT-trimethoprim/sulfamethoxazole, CIP-ciprofloxacin, PRL-piperacillin, C-chloramphenicol, AMC-amoxicillin/clavulanic acid, CXM-cefuroxime, NF-nitrofurantoin;

Table S10. Genotypic and phenotypic characterization of *E. coli* isolates from food (FOOD)

No./ Source of meet	Strains	Ph	Virulence/profiles								Resistance/patterns
			Adhesins			Toxins		Miscellanous			
			fimH	*papC*	*iha*	*vat*	*pic*	*irp2*	*iss*	*usp*	
1/h	FOOD10	B1	■							■	AMP/TE
2/h	FOOD11	F	■			■	■	■	■	■	AMP/TE/CIP
3/h	FOOD16	U		■				■		■	CIP
4/h	FOOD2	B1	■							■	
5/h	FOOD20	B1	■	■			■	■	■	■	CIP
6/h	FOOD21	A	■	■			■	■		■	
7/h	FOOD22	A	■	■			■	■		■	
8/h	FOOD28	B1	■	■	■		■		■	■	AMP/TE/CIP
9/h	FOOD29	U	■	■			■		■	■	
10/h	FOOD3	F	■			■	■	■	■	■	AMP/TE
11/h	FOOD31	A	■	■			■		■		
12/h	FOOD33	A	■	■			■	■	■		AMP/TE/SXT/CIP
13/h	FOOD34	E		■			■		■	■	AMP/TE/SXT/CIP
14/h	FOOD38	U	■	■			■	■	■		
15/h	FOOD41	B1	■	■			■	■	■		AMP/TE/SXT/CIP/C
16/h	FOOD42	F	■	■		■	■	■	■		TE/CIP
17/h	FOOD44	A	■	■			■	■	■		AMP/TE/SXT/CIP
18/h	FOOD45	A	■					■	■		AMP/TE/SXT/CIP
19/h	FOOD46	A	■	■	■		■	■	■		AMP/SXT
20/h	FOOD48	A	■	■			■		■	■	AMP/TE/SXT/CIP
21/h	FOOD5	A									
22/h	FOOD6	A								■	
n(%)	22(57.9)		18	15	2	3	16	13	15	13	MDR(n=8)

23/p	FOOD115N	A	■	■			■	■	■		AMP/TE/SXT/CIP
24/p	FOOD12	A	■							■	
25/p	FOOD13	A	■				■	■		■	
26/p	FOOD15	A	■						■	■	
27/p	FOOD18	A	■	■			■	■	■	■	
28/p	FOOD19	A	■	■			■	■		■	
29/p	FOOD25	A	■	■				■	■	■	
30/p	FOOD27	B1	■	■	■		■	■	■	■	
31/p	FOOD36	F	■	■			■		■		AMP/TE/SXT/CIP
32/p	FOOD43	A	■	■			■	■	■		AMP/TE
n(%)	10(26.3)		10	7	1	0	7	7	7	7	MDR(n=2)
33/t	FOOD23	U	■	■		■	■	■		■	
34/t	FOOD24	A	■	■					■	■	AMP/TE/SXT/CIP
35/t	FOOD30	B1	■	■	■		■		■	■	AMP
36/t	FOOD35	B1	■	■	■		■		■		AMP/TE/SXT/CIP/PRL/AK
n(%)	4(10.5)		4	4	2	1	3	1	3	3	MDR(n=2)
37/b	FOOD119N	A	■	■	■		■		■		TE/SXT/CIP
38/b	FOOD19S	A	■	■	■		■	■	■		AMP/TE/SXT/CIP
n(%)	2(5.3)		2	2	2	0	2	1	2	0	MDR(n=2)
Total (n)%	38 (100)		34 (89.5)	28 (73.7)	7 (18.4)	4 (10.5)	28 (73.7)	22 (57.9)	27 (71.1)	23 (60.5)	MDR total n=14 (36.8)

Type of meat: h–chicken, p–pork, t–turkey, b–beef;

Ph – Phylogroup; U – Unknown

Note: MDR–multidrug resistance; AMP-ampicillin, TE-tetracycline, SXT-trimethoprim/sulfamethoxazole, CIP-ciprofloxacin, PRL-piperacillin, C-chloramphenicol, AMC-amoxicillin/clavulanic acid, AK-amikacin;

Table S11. Genotypic and phenotypic characterization of *E. coli* isolates from poultry farms (Poultry farm – AFEC))

No./ Farming system	Strains	Ph	Virulence/profiles								Resistance/patterns
			Adhesins			Toxins		Miscellanous			
			fimH	*papC*	*iha*	*vat*	*pic*	*irp2*	*iss*	*usp*	
1/f	JA01	F	■	■				■	■		
2/f	JA02	A	■	■			■	■	■	■	
3/f	JA03	A	■	■			■	■	■	■	
4/f	JAN1	A	■					■	■		
5/f	KA01	A	■	■							
6/f	KA02	B1	■	■			■	■	■	■	
7/f	KB01	A	■							■	
8/f	KB02	A	■	■			■	■	■	■	
n(%)	8(18.2)		8	6	0	0	4	6	6	5	MDR(n=0)
9/df	PT101	B1	■						■	■	AMP/GE
10/df	PT102	B1	■				■	■	■	■	AMP
11/df	PT1K2	B1	■				■	■	■	■	AMP/TE/SXT/CIP/C
12/df	PT1N1	A	■								AMP/TE/PRL
13/df	PT1N2	B1	■				■	■	■	■	AMP/TE/PRL
14/df	PT201	D	■								
15/df	PT2K2	D	■	■		■	■	■	■	■	
16/df	PT3K1	D	■							■	
17/df	PT3K2	E	■	■			■	■	■	■	AMP/TE/CIP/C
n(%)	9(20.5)		9	2	0	1	5	5	6	7	MDR(n=4)
18/d	CJ01	B1	■								
19/d	CJ02	A	■	■		■	■	■	■	■	AMP/TE/PRL
20/d	FD101	A	■				■	■			
21/d	FD201	A	■					■			AMP/CIP/C
22/d	FD202	D	■	■			■	■	■	■	TE/SXT/ESBL+
23/d	FD301	A	■					■	■		AMP/TE/SXT/CIP

24/d	FD3K2	B2	■				■	■	■	■	TE/SXT/CIP/C/ESBL+
25/d	PW01	D	■						■		AMP/TE/SXT
26/d	PW02	C	■	■			■	■	■	■	
n(%)	9(20.5)		9	3	0	1	5	7	6	4	MDR(n=6)
27/c	BW01	B1		■					■		AMP
28/c	BW02	B2	■	■			■	■	■	■	
29/c	BW03	B1	■				■	■	■	■	
30/c	CM01	B1						■	■		AMP/SXT/C
31/c	CM02	D	■	■			■	■	■	■	AMP/TE/SXT/PRL
32/c	CMK2	B1	■	■			■	■	■		AMP/TE/SXT/PRL
33/c	DP01	C	■	■							
34/c	DP02	B1	■	■	■		■	■	■	■	TE/SXT
35/c	DP03	A	■				■	■	■	■	AMP
36/c	MJ01	C	■	■				■			
37/c	MJ02	A	■	■			■	■	■		
38/c	WW01	B1	■					■			AMP
39/c	WW02	A	■	■			■	■	■	■	
40/c	ZMK1M	U	■								
41/c	ZMN1M	B2	■	■				■	■	■	
42/c	ZMN2M	F	■	■		■	■	■	■	■	
43/c	ZMN2S	F	■	■			■	■	■	■	
44/c	ZMN3M	D	■			■	■	■	■	■	AMP/TE/SXT/CIP/C
n(%)	18(40.9)		16	12	1	2	11	15	14	10	MDR(n=4)
Total n(%)	44 (100)		42 (95.5)	23 (52.3)	1 (2.3)	4 (9.1)	25 (56.8)	33 (75.0)	32 (72.7)	26 (59.1)	MDR total n=14 (31.8%)

Farming system: c – cage, d –deep litter, f – free-range, df – deep litter and free-range;

Ph – Phylogroup; U – Unknown

Note: MDR–multidrug resistance; AMP-ampicillin, TE-tetracycline, SXT-trimethoprim/sulfamethoxazole, CIP-ciprofloxacin, PRL-piperacillin, C-chloramphenicol, AMC-amoxicillin/clavulanic acid, CXM-cefuroxime, GE-gentamicin, CTX-cefotaxime;

Table S12. ERIC-PCR profiles and STs (MLST) with the highest number of similarities among *E. coli* isolates

Cluster/ ERIC	Strains/ MLST (ST)	Virulence/profiles								Resistance/patterns
		fimH	*usp*	*irp2*	*vat*	*papC*	*pic*	*iss*	*iha*	
EI	WW01/ST1049	■		■						AMP
	FOOD 28(h)/U	■	■			■	■	■	■	AMP/TE/CIP
EII	KA01/U	■				■				-
	FOOD 29(h)/U	■	■			■	■	■		-
EIII	CM02/U	■	■	■		■	■	■		AMP/TE/SXT/PRL
	DP01/U	■				■				-
	UTI 16/ST69	■	■	■						AMP/SXT
EIV	CJ01/ST2534	■								-
	FOOD 23(t)/U	■	■	■	■	■	■			-
EV	UTI 28/U	■	■	■	■	■				AMP
	FOOD 21(h)/ST536	■	■	■		■	■			-
EVI	PT1N1/U	■								AMP/SXT
	FOOD 25(p)/ ST10888	■	■	■		■		■		-
EVII	MJ01/U	■		■		■				-
	FOOD 35(t)/U	■				■	■	■	■	AMP/TE/CIP/ SXT/PRL/AK
EVIII	WW02/ST212	■	■	■		■	■	■		-
	FOOD 10(h)/ ST6073	■	■							AMP/TE
EIX	FD202/ST5451	■	■	■		■	■	■		TE/SXT/ESBL+
	FD3K2/ST624	■	■	■			■	■		TE/CIP/SXT/C/ESBL+

	UTI 9/ST354		■	■	■			■		AMP/TE/SXT
	UTI 31/ST404	■	■	■	■	■	■			AMP/SXT
EX	UTI 7/U	■	■	■	■			■		AMP/TE/CIP/SXT
	FOOD 38(h)/U	■		■		■	■	■		-
EXI	UTI 42/U	■	■	■	■	■	■	■		AMP/TE/CIP/NF/SXT
	FOOD 19s(b)/ ST5295	■		■		■	■	■	■	AMP/TE/CIP/SXT

Note: ■ – positive result (presence of gene); ST – sequence type (MLST); U – unknown ST;
AMP-ampicillin, TE-tetracycline, SXT-trimethoprim/sulfamethoxazole, CIP-ciprofloxacin, PRL-piperacillin, C-chloramphenicol, AMC-amoxicillin/clavulanic acid, CXM-cefuroxime, NF-nitrofurantoin, CTX-cefotaxime, AK-amikacin;
Type of meat: h-chicken, p-pork, t-turkey, b-beef;

Table S13. The allelic profiles of seven housekeeping genes: *adk, gyrB, fumC, icd, mdh, purA* and *recA* (variants of alleles marked in blue, which repeat among the tested strains) of 25 *E. coli* strains isolated from poultry farms, food, and patients with UTI symptoms. The sequence types (ST) highlighted in red (seven housekeeping genes) and sequence types highlighted in green (six housekeeping genes)

ID	MLST (7)	MLST (6)	*icd*	*fumC*	*gyrB*	*purA*	*adk*	*mdh*	*recA*
FOOD 10(h)	U	ST6073	619	19	14	12	842	11	2
FOOD 19s(b)	ST5295	ST5295	1	126	5	8	64	8	6
FOOD 21(h)	U	ST536	95	107	1	8	502	69	20
FOOD 23(t)	U	U	1	7	5	18	842	8	2
FOOD 25(p)	ST10888	ST10888	1273	11	5	8	112	8	86
FOOD 28(h)	U	U	18	19	33	122	457	9	6
FOOD 29(h)	U	U	8	11	4	8	232	8	49
FOOD 35(t)	U	U	26	65	32	8	502	9	2
FOOD 38(h)	U	U	613	11	4	8	623	8	49
UTI 7	U	U	1	65	5	13	457	9	6
UTI 9	ST354	ST354	29	88	78	58	85	59	62
UTI 16	ST69	ST69	6	35	27	5	21	5	4
UTI 28	U	U	13	40	311	28	53	36	29
UTI 31	ST404	ST404	14	14	10	7	14	17	74
UTI 42	U	U	8	11	135	8	864	8	2
DPO1(c)	U	U	8	81	4	8	623	12	2
CMO2(c)	U	U	6	35	27	5	864	5	4
FD3K2(d)	ST624	ST624	96	4	87	58	92	70	91
FD202(d)	ST5451	ST5451	342	3	234	5	35	5	302
CJO1(d)	ST2534	ST2534	88	19	15	193	137	7	7
KAO1(f)	U	U	88	4	15	8	457	80	14
MJO1(c)	U	U	564	1054	60	8	174	509	677
PT1N1(df)	U	U	1	65	5	13	457	9	6
WWO1(c)	U	ST1049	16	4	14	5	46	24	14
WWO2(c)	U	ST212	18	29	4	8	502	11	6

Note: U – unknown ST; Farming system: c – cage, d –deep litter, f – free-range, df – deep litter and free-range; Type of meat: h–chicken, p–pork, t–turkey, b–beef;

Table S14. STs (MLST) and phylogroups (Clermont) among 25 *E. coli* isolates

MLST/ Branch	Strains/ ST/Ph	Virulence/profiles								Resistance/patterns
		fimH	*usp*	*irp2*	*vat*	*papC*	*pic*	*iss*	*iha*	
I	WW02/ST212/**A**	■	■	■		■	■	■		-
	FOOD 21(h)/ ST536/**A**	■	■	■		■	■			-
	FOOD 35(t)/U/**B1**	■				■	■	■	■	AMP/TE/CIP/ SXT/PRL/AK
	DP01/U/**C**	■				■				-
II	PT1N1/U/**A**	■								AMP/TE/PRL
	KA01/U/**A**	■				■				-
	UTI 7/U/**B1**	■	■	■	■			■		AMP/TE/CIP/SXT
	CJ01/ST2534/**B1**	■								-
	FOOD 28(h)/U/**B1**	■	■			■	■	■	■	AMP/TE/CIP
III	UTI 9/ST354/**B2**		■	■	■			■		AMP/TE/SXT
	FD3K2/ST624/**B2**	■	■	■			■	■		TE/CIP/SXT/C/ESBL+
	UTI 28/U/**F**	■	■	■	■	■				AMP
	UTI 31/ST404/**B2**	■	■	■	■	■	■			AMP/SXT
IV	FD202/ ST5451/**D**	■	■	■		■	■	■		TE/SXT/ESBL+
	CM02/U/**D**	■	■	■		■	■	■		AMP/TE/SXT/PRL

	UTI 16/ST69/**D**	■	■	■						AMP, SXT
	WW01/ST1049/**B1**	■		■						AMP
	FOOD 10(h)/ ST6073/**B1**	■	■							AMP/TE
V	FOOD 38(h)/U/**U**	■		■		■	■	■		-
	FOOD 29(h)/U/**U**	■	■			■	■	■		-
	UTI 42/U/**C**	■	■	■	■	■	■	■		AMP/TE/CIP/NF/SXT
	FOOD 23(t)/U/**U**	■	■	■	■	■	■			-
	FOOD 19s(b) /ST5295/**A**	■	■		■		■	■	■	AMP/TE/CIP/SXT
	FOOD 25(p)/ ST10888/**A**	■	■	■		■		■		-

Note: ■ – presence of gene; ST – sequence type (MLST); Ph – Phylogroup;
U – unknown; AMP-ampicillin, TE-tetracycline, SXT-trimethoprim/sulfamethoxazole, CIP-ciprofloxacin, PRL-piperacillin, C-chloramphenicol, AMC-amoxicillin/clavulanic acid, CXM-cefuroxime, NF-nitrofurantoin, CTX-cefotaxime, AK-amikacin;
Type of meat: h-chicken, p-pork, t-turkey, b-beef;

Table S15. Statistical association between VGs of *E. coli* isolates derived from poultry farms (n=44) – Phi coefficient

fimH	*papC*	*iha*	*irp2*	*iss*	*usp*	*vat*	*pic*
fimH	0.01	0.03	0.12	0.13	0.26	0.07	0.25
	papC	0.14	0.29	0.33*	0.22	0.14	0.36*
		iha	0.09	0.09	0.13	0.04	0.13
			irp2	0.59*	0.37*	0.18	0.66*
				iss	0.53*	0.19	0.60*
					usp	0.26	0.67*
						vat	0.27
							pic

Phi coefficient values:

Very strong associations between variables φ >0.7; strong associations 0.5<φ<0.7;
moderate associations 0.3<φ<0.5; weak associations φ<0.3

Table S16. Statistical association between VGs of *E. coli* isolates derived from UTI (n=50) - Phi coefficient

fimH	*papC*	*iha*	*irp2*	*iss*	*usp*	*vat*	*pic*
fimH	0.24	0.09	0.03	0.21*	0.02	0.08	0.13
	papC	0.09	0.34*	0.28*	0.24	0.27	0.27
		iha	0.13	0.02	0.09	0.09	0.18
			irp2	0.08	0.70*	0.11	0.02
				iss	0.21	0.01	0.03
					usp	0.24	0.13
						vat	0.46*
							pic

Phi coefficient values:
Very strong associations between variables φ >0.7; strong associations 0.5<φ<0.7
moderate associations 0.3<φ<0.5; weak associations φ<0.3

Table S17. Statistical association between VGs of *E. coli* isolates derived from food (n=38) - Phi coefficient

fimH	*papC*	*iha*	*irp2*	*iss*	*usp*	*vat*	*pic*
fimH	0.18	0.16	0.23	0.35*	0.10	0.12	0.38*
	papC	0.28	0.22	0.41*	0.24	0.18	0.59*
		iha	0.14	0.30	0.17	0.16	0.28
			irp2	0.04	0.14	0.29	0.34*
				iss	0.40*	0.03	0.41*
					usp	0.10	0.24
						vat	0.20

pic

Phi coefficient values:
Very strong associations between variables $\varphi > 0.7$; strong associations $0.5 < \varphi < 0.7$
moderate associations $0.3 < \varphi < 0.5$; weak associations $\varphi < 0.3$

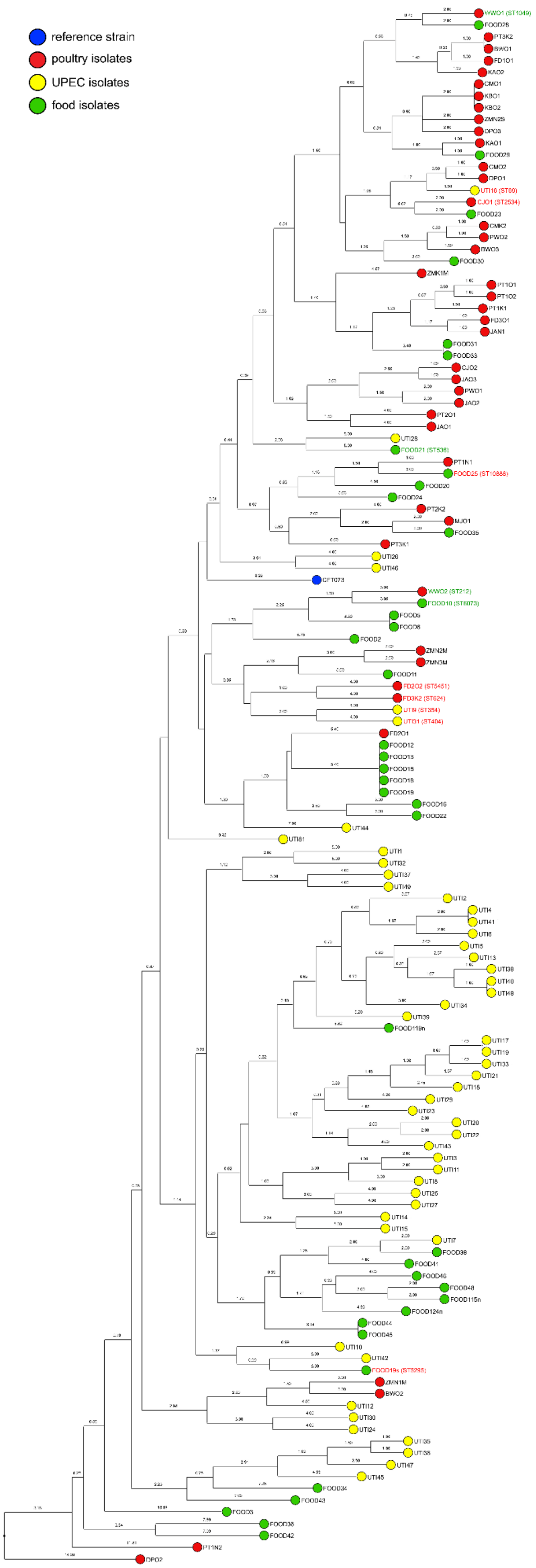
reference strain
poultry isolates
UPEC isolates
food isolates

Figure S1. Dendrogram generated from ERIC-PCR banding pattern of 132 *E. coli* isolated from poultry farms, food and patients with UTI symptoms. The process was performed in a volume of 25 μL, including 2.5 μL of 10x Taq buffer, 2.5 μL of each dNTP Mix, 0.6 μL of forward and reverse primers, 1 μg of template DNA, 0.62 U of DreamTag DNA polymerase, and nuclease-free water. The reaction products were isolated with 1% agarose gel electrophoresis (Amresco) and visualized with GelDoc XR (Biorad). The similarity analysis was performed with Dice coefficient and UPGMA method.
